# Analysis of circadian pattern reveals tissue-specific alternative transcription in leptin signaling pathway

**DOI:** 10.1186/1471-2105-8-S7-S15

**Published:** 2007-11-01

**Authors:** Andrey A Ptitsyn, Jeffrey M Gimble

**Affiliations:** 1 Experimental Obesity Laboratory, Louisiana State University Pennington Biomedical Research Center, 6400 Perkins Rd., Baton Rouge, LA 70808, USA; 2 Stem Cell Laboratory, Louisiana State University System, Pennington Biomedical Research Center, 6400 Perkins Rd., Baton Rouge, LA 70808, USA

## Abstract

**Background:**

It has been previously reported that most mammalian genes display a circadian oscillation in their baseline expression. Consequently, the phase and amplitude of each component of a signal transduction cascade has downstream consequences.

**Results:**

Here, we report our analysis of alternative transcripts in the leptin signaling pathway which is responsible for the systemic regulation of macronutrient storage and energy balance. We focused on the circadian expression pattern of a critical component of the leptin signaling system, suppressor of cytokine signaling 3 (SOCS3). On an Affymetrix GeneChip 430A2 microarray, this gene is represented by three probe sets targeting different regions within the 3' end of the last exon. We demonstrate that in murine brown adipose tissue two downstream 3' probe sets experience circadian baseline oscillation in counter-phase to the upstream probe set. Such differences in expression patterns are a telltale sign of alternative splicing within the last exon of SOCS3. In contrast, all three probe sets oscillated in a common phase in murine liver and white adipose tissue. This suggests that the regulation of SOCS3 expression in brown fat is tissue specific. Another component of the signaling pathway, Janus kinase (JAK), is directly regulated by SOCS and has alternative transcript probe sets oscillating in counter-phase in a white adipose tissue specific manner.

**Conclusion:**

We hypothesize that differential oscillation of alternative transcripts may provide a mechanism to maintain steady levels of expression in spite of circadian baseline variation.

## Background

Circadian or approximately daily rhythms occur throughout nature. Entrained by the daily light/dark cycle, feeding behavior, sleeping pattern and/or other environmental and physiological cues, gene expression patterns also demonstrate daily oscillations. Until recently, it has been widely accepted that up to 10–15 per cent of all genes are expressed following a circadian rhythm [1], generated by a negative feedback molecular oscillator, aka basic circadian clock. In mammals, the central circadian clock is synchronized to the daily light/dark cycle and is located in the Suprachiasmic Nucleus (SCN) of the hypothalamus. However, it has been demonstrated that circadian clocks are active in peripheral tissues [2-5]. The peripheral clocks are synchronized through sympathetic outputs and the controlled secretion of circulating glucocorticoids, melatonin, and other mediators. Our recent publications suggest that the scale and role of circadian oscillation in gene expression has been underestimated [6,7]. We have presented evidence of baseline gene expression oscillations that exceed the accepted 10–15% and involve nearly all expressed genes. One of the most important implications of this discovery is related to the way we model, visualize, and understand biological pathways. Since virtually every component of each biological pathway is oscillating, the timing of regulation, interaction or signal transduction is critically important as well as the phase of oscillation at which a particular event takes place.

## Results

To illustrate the concept of oscillation within a biological pathway we have chosen the leptin pathway as an example. Leptin is an important regulator of the energy balance in the organism and contributes to the sensation of satiety after a meal. Leptin-deficient mice are prone to overeating, excessive weight gain, and the development of obesity. A similar phenotype is observed in the case of leptin-deficient humans. Leptin is highly expressed in adipose tissue and secreted into the bloodstream. The leptin receptor (LEPR) is actively expressed in the liver. Upon ligand-receptor activation, a signal is transmitted through the Janus Kinase (JAK) and STAT3 cascade, which are shared in common with related signal transduction pathways. SOCS3, whose transcription is activated by STAT3, represses both JAK and LEPR and serves to attenuate the signal.

**Figure 1:**
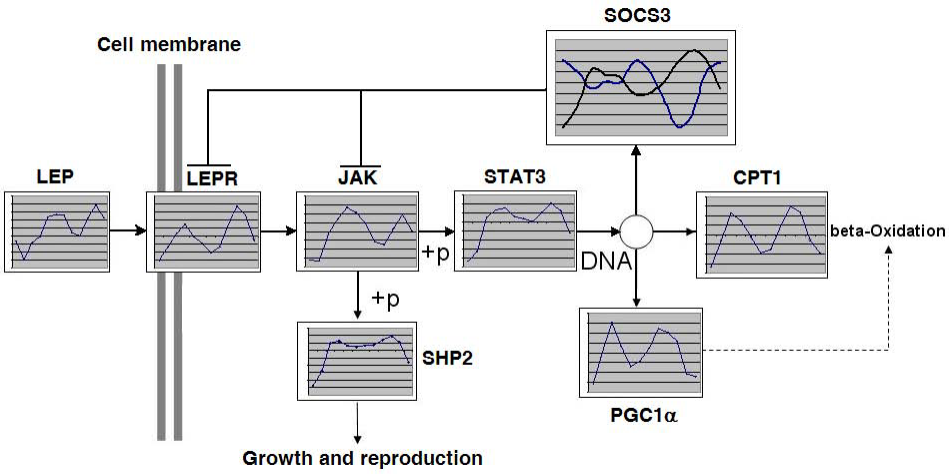
**Circadian oscillation in leptin signaling pathway in mouse brown fat**. This is a fragment of the KEGG pathway with superimposed plots of circadian expression profiles for the essential components of the pathway. Every gene involved in leptin signaling demonstrates explicit circadian pattern in baseline oscillation. All except one oscillate in the same phase. Leptin itself is actively expressed in brown adipose tissue and its intensity oscillates with a phase shift, slightly behind leptin receptor (LEPR) and other components of leptin signaling. Remarkably, suppressor of cytokine signal (SOCS3) has two alternative transcripts oscillating in counter-phase.

This leptin signal transduction pathway is well studied and can be found in a number of private and public databases. We have extracted the description along with the cartoon illustrating the leptin pathway from the Kyoto Encyclopedia of Genes and Genomes (KEGG)[8], We have scaled, smoothed, and assigned to most likely phase class the data sets with microarray expression profiles from the previously published studies (see Methods). By overlapping the gene expression profile for a particular tissue with the general leptin signaling cartoon, we produced a diagram illustrating the relative abundance of transcripts for each component of the pathway at a given time of the day (Figure 1).

As shown on Figure 1 in brown adipose tissue both leptin and leptin receptor are expressed in a circadian pattern with two peaks over the two-day observation period. The acrophase of leptin receptor is slightly ahead of the leptin acrophase, indicating increase in receptor production in anticipation of the signal. All other components of the leptin signaling pathway shown on Figure 1 have expression levels synchronized with the expression of the leptin receptor, i.e. they oscillate in the same phase.

However, SOCS3 is the exception to this pattern. In Figure 1, SOCS3 expression is present in two separate and contradictory plots oscillating in opposite phases; the acrophase (time of the highest point) of one profile corresponds to the bathyphase (time of the lowest point) of the other at an equivalent time point. In Figure 1, we have plotted 2 of the 3 probesets representing SOCS3 on the Affymetrix Mouse Expression Genechip. The observation that alternative probesets interrogating expression levels of the same gene could demonstrate such a contrast was novel and called for further investigation.

**Figure 2:**
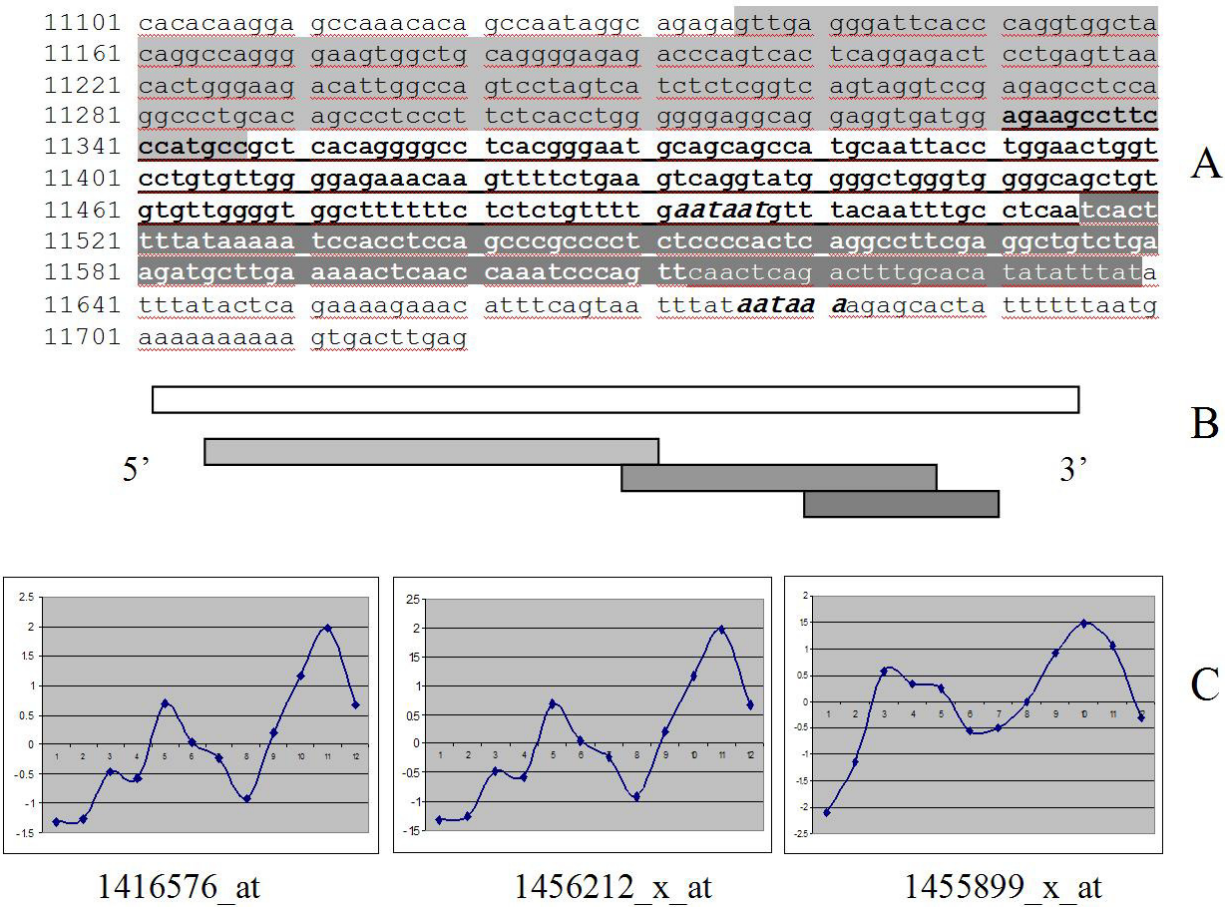
**Overlapping target sequences for Affymentrix probesets in 3'UTR of murine SOCS3 gene**. Affymetrix mouse gene expression GeneChip 430 has 3 probesets for SOCS3 gene derived from 3 target sequences. These overlapping stretches of 3'UTR are highlighted with grey, dark gray and underlining on panel A. Panel B shows a map of overlapping target sequences (gray) in SOCS3 3'UTR (white). The probesets 1416576_at, 1456212_x_at and 1455899_x_at demonstrate individual expression pattern. Three plots of circadian profiles for these probesets on panel C are arranged in the same 5' to 3' order as overlapping target sequences on panel B. The last two probesets oscillate in the counter-phase to the first one.

We extracted the target sequences for the individual probe sets represented on the GeneChip from the supporting materials provided by Affymetrix through the Developer Network. Then, we used these target sequences as queries in a BLAST search against the non-redundant set of nucleotide sequences and mouse ESTs. The target sequences were also aligned to the genomic sequence corresponding to the murine SOCS3 gene. The results of this mapping are shown in Figure 2.

All three Affymetrix probesets from SOCS3 were derived from the alternative consensi that result from alignment of murine ESTs to the genomic sequence (Additional File 1). All three target sequences from which the probesets are derived represent overlapping stretches of the 3' UTR of the SOCS3 gene. Comparing the relative positions of the target sequences and alignment of the ESTs from which they were derived, we came to the conclusion that the Affymetrix probesets represented alternative transcripts of different length, consistent with alternative polyadenylation. The presence of two potential polyadenylation signals within the 3' UTR of SOCS3 gene targeted by the probesets supports this hypothesis. The 3' polyadenylation signal has a canonic AATAAA sequence starting at position 11675. 5' to the canonic signal at position 11492 there is an alternative sequence AATAAT, also described in the literature as a potential PolyA signal [9]. The primary Affymetrix probeset 1416576_at targets the most 5' part of the 3' UTR, common for both alternative transcripts. The most downstream probeset 1455899_x_at targets the extreme 3' end specific to the longer version of SOCS3 transcript. The third probeset representing 1456212_x_at targets the area up- and downstream of the weaker alternative polyA signal. The target sequence for this probeset is contained within the longer transcript completely, but only partially present in the shorter alternative version.

If our conclusion about Affymetrix probesets representing alternatively polyadenylated transcripts of different length is true, the middle of three probesets containing probes for both transcripts should demonstrate a mixed pattern. Indeed, as presented on Figure 2, expression profile of the intermediate probeset 1456212_x_at looks more erratic than the other two profiles. This profile also shows a remarkable similarity to the arithmetic sum of the expression profiles for the flanking 1416576_at and 1455899_x_at probesets.

The described phenomenon of alternative transcripts with contrasting oscillation patterns is not unique to SOCS3. We have analyzed the oscillation pattern in the components of the same pathway in different tissues. In liver we found no evidence of probesets with different phase of oscillation. Some of the probesets corresponding to the same transcript demonstrate different degree of noise, but allow no conclusive statement regarding the phase of oscillation. In white adipose tissue SOCS3 probesets also show no evidence of alternative oscillating pattern. However, the other component of the same pathway, JAK, has alternative probesets oscillating in counter-phase, just like SOCS3 in the brown adipose tissue.

## Discussion

The notion that all genes being expressed in an oscillating manner has a few important implications. First, a steady line gene expression, i.e. a constant level of transcript abundance over time, is an abstraction for which we see no evidence in experimental data. If some transcripts are to be maintained at a certain level of abundance, this level can be constant only in relation to some other transcripts, which also means that these genes have to oscillate in the same phase. Even so called "housekeeping" genes, abundant and essential for the survival of a cell, in fact do experience daily oscillation of the baseline transcription. Affymetrix chips can register this oscillation because relative intensity of expression for each probeset is normalized to the mean expression level of the control probes. Control probes include a large number of well studied "housekeeping" genes. However, the GeneChip technology has been designed with no knowledge and thus no reservation for periodic gene expression. Control probesets represent all possible phases and their number is large enough to have all possible phases represented approximately equally. All oscillations are averaged out and disappear when control sets are pooled *in silico *to normalize the expression of the rest of the chip. This resulting steady line is artificial, but solid enough to make all other phases of oscillation detectable. The second important implication is that the negative-feedback molecular clock is unlikely to be the main generator of all oscillations. It is hard to see a reason why genes from the oxidative phosphorylation pathway should be regulated by a circadian rhythm. But the opposite is more likely: multiple publications point that oxidative phosphorylation pathway is a natural oscillator and time-separation of biochemical reactions is essential for the aerobic metabolism [10-13]. Indeed in murine tissue samples we find genes related to oxidative phosphorylation expressed in the most clear noise-free circadian rhythm. It is likely that in higher eukaryotes the respiratory cycle is synchronized with the metabolic cycle and thus expression patterns of all other genes are modulated by the oscillation of the main energy production cycle.

Microarrays are designed to provide a snapshot of relative abundance of thousands of transcripts at a time. The abundance of transcripts depends on many factors, of which one of the most important is the turnover rate of a particular mRNA. The ratio between the rate of transcription, maturation, localization, and the rate of degradation of mRNA determines the number of active copies available for translation. The structure of 3' UTR determines localization and stability of the transcript. The great majority of genes are represented by a mixed population of transcripts resulting from the alternative processing during mRNA maturation, while alternative polyadenylation is arguably the most common cause for such variation. It has been previously reported that oscillating genes can have alternative transcripts of different length [14,15]. Different length of 3' UTR results in a different turnover rate of PER3 protein, one of the key elements of the circadian molecular clock in mammals. Changes in PER3 turnover may result in accumulation of available PER3 mRNA and increased rates of protein synthesis, which leads to alteration of sleeping patterns, feeding behavior, and other periodic activities. Since alternative transcripts of SOCS3 are slightly different lengths and 3' UTR structure are very common it is logical to suggest that alternative transcripts may have different observed oscillating properties, resulting from the different turnover rates of alternative transcripts.

The observed pattern of a longer transcript oscillating in an opposite phase to the shorter version may have a special function. We hypothesize that this phenomenon may represent the cellular mechanism generating a "steady line" non-oscillating production of an important protein. Both SOCS3 and JAK are essential elements of a number of signal transduction pathways that may not be synchronized. Signal transduction events may take place at different time and a steady number of mature JAK and SOCS3 proteins have to be maintained in anticipation of such signals. A recent publication suggests that high abundance of SOCS3 transcripts is responsible for leptin resistance in Diet-Induced Obesity (DIO) [16]. We believe that shifting a balance between shorter and longer lived transcripts may contribute to the molecular mechanism for reversible leptin resistance reported by Cowley et al. [16].

If respiratory oscillation is a common primordial rhythm of all eukaryotes, the eukaryotic cell always exists in a volatile state with no absolute expression levels, each gene is oscillating and changes the transcript abundance in cytoplasm only relative to the other oscillating genes. In this condition generation of a protein whose abundance is invariant is a result of adaptive evolution. In other words, continuous oscillation is a default state and the steady abundance of a protein is a functional adaptation. In the case of JAK and SOCS3, we believe that the different turnover rate of two alternative transcripts serves the purpose of overcoming the natural oscillation of transcription rate and contributes to the maintenance of a steady production of these important proteins. One transcript is more stable, while the other is transcribed at a higher rate; relative abundance of one increases when abundance of the other declines. The longer transcript is not necessarily the most stable: it may contain a signal responsible for rapid transcript decay mediated by short non-coding RNA. Further experimental study will be required to determine individual turnover rates of the alternative SOCS3 transcripts and potential for tipping the balance of their relative abundance in order to normalize leptin signaling through the JAK-STAT3 pathway.

## Conclusion

We report the discovery of alternative transcripts demonstrating a contrast oscillating pattern. This pattern is tissue-specific. SOCS3 transcripts of different length resulting from the alternative polyadenylation in 3' UTR area are found to be oscillating in the opposite phase in murine brown adipose tissue, but not in white adipose tissue or liver. The other component of the same signal transduction cascade, JAK has alternative transcripts oscillating in counter-phase in white adipose tissue, but not in liver or brown adipose tissue. We propose a hypothesis that alternative transcripts in counter-phase represent the evolutionary developed compensation for oscillating transcription rate. This function provides a steady non-oscillating abundance of essential proteins in eukaryotic cell.

## Methods

### Murine circadian gene expression data

We have completed independent circadian studies in AKR/J mice acclimated to a 12 hr light: 12 hr dark cycle, harvesting sets of 3–5 mice at 4 hr intervals in duplicates over a 24 hr period [4]. Total RNA samples from inguinal (iWAT) white adipose tissue, brown adipose tissue (BAT), and liver have been assayed by Affymetrix microarrays. A few genes have been selected for validation with RT-PCR for the expression profile of representative circadian rhythm genes in all 3 tissues. The transcriptomic data set contained over 22,000 gene expression profiles for each of 3 different tissues. In the current study, we have used only the murine liver data. Since each time point was sampled twice, the following Fourier transform for each profile can be re-arranged into a short time series that represents two complete circadian cycles. Smoothing and median subtraction procedure has been applied to all data sets.

### Data pre-processing

Profiles have been smoothened by a 3^rd ^degree polynomial procedure and median-subtracted. For smoothing we use seven-point Savitzky-Golay algorithm [17]. To take advantage of all points in the time series a single-pass smoothing has been applied in a circular manner, with the last points contributing to smoothing the starting points. For better compatibility, the same smoothing and median subtraction procedure has been applied to all data sets.

### Phase classification

We have assigned phase to each expression time series by computing cross-correlation

R(f)=∑0N−1(xi−x¯)(yf−y¯)∑0N−1(xi−x¯)(yi−y¯)
 MathType@MTEF@5@5@+=feaafiart1ev1aaatCvAUfKttLearuWrP9MDH5MBPbIqV92AaeXatLxBI9gBaebbnrfifHhDYfgasaacH8akY=wiFfYdH8Gipec8Eeeu0xXdbba9frFj0=OqFfea0dXdd9vqai=hGuQ8kuc9pgc9s8qqaq=dirpe0xb9q8qiLsFr0=vr0=vr0dc8meaabaqaciaacaGaaeqabaqabeGadaaakeaacqWGsbGucqGGOaakcqWGMbGzcqGGPaqkcqGH9aqpdaWcaaqaamaaqadabaWaaeWaaeaacqWG4baEdaWgaaWcbaGaeeyAaKgabeaakiabgkHiTiqbdIha4zaaraaacaGLOaGaayzkaaWaaeWaaeaacqWG5bqEdaWgaaWcbaGaemOzaygabeaakiabgkHiTiqbdMha5zaaraaacaGLOaGaayzkaaaaleaacqaIWaamaeaacqWGobGtcqGHsislcqaIXaqma0GaeyyeIuoaaOqaamaaqadabaWaaeWaaeaacqWG4baEdaWgaaWcbaGaemyAaKgabeaakiabgkHiTiqbdIha4zaaraaacaGLOaGaayzkaaWaaeWaaeaacqWG5bqEdaWgaaWcbaGaemyAaKgabeaakiabgkHiTiqbdMha5zaaraaacaGLOaGaayzkaaaaleaacqaIWaamaeaacqWGobGtcqGHsislcqaIXaqma0GaeyyeIuoaaaaaaa@5A28@

where *x *is a gene expression time series of *N *points and *y *is an artificially generated profile of ideal cosine function yi=cos⁡(2πp*i)
 MathType@MTEF@5@5@+=feaafiart1ev1aaatCvAUfKttLearuWrP9MDH5MBPbIqV92AaeXatLxBI9gBaebbnrfifHhDYfgasaacH8akY=wiFfYdH8Gipec8Eeeu0xXdbba9frFj0=OqFfea0dXdd9vqai=hGuQ8kuc9pgc9s8qqaq=dirpe0xb9q8qiLsFr0=vr0=vr0dc8meaabaqaciaacaGaaeqabaqabeGadaaakeaacqWG5bqEdaWgaaWcbaGaemyAaKgabeaakiabg2da9iGbcogaJjabc+gaVjabcohaZnaabmaabaWaaSaaaeaacqaIYaGmiiGacqWFapaCaeaacqWGWbaCaaGaeiOkaOIaemyAaKgacaGLOaGaayzkaaaaaa@3CD1@, where p is the number of time points in a complete circadian cycle, for example 6 in Zvonic et al. [4] data set. To account for all phases the artificial cosine curve profile has been regenerated with a phase shift by 1 time point. The highest correlation among all possible phase shifts was assigned as the most probable phase. The significance of periodicity was not assessed at this point, it was done separately by three independent procedures described above. All expression profiles were sorted (classified) first by the assigned phase then by ascending p-value estimated by one of the described algorithms.

## Competing interests

The authors declare that they have no competing interests.

## Authors' contributions

AAP has developed the algorithms, the software and performed data analysis and algorithm testing and benchmarking. AAP and JMG wrote the paper.

## Supplementary Material

Additional file 1 Graphical overview of the BLAST alignment of mouse genome fragment containing SOCS3 sequence and homologous mouse ESTs. Click here for file
