# Supplementary figures and images for: Analysis of circadian pattern reveals tissue-specific alternative transcription in leptin signaling pathway
Source: BMC Bioinformatics. 2007 Nov 1;8(Suppl 7):S15. doi: 10.1186/1471-2105-8-S7-S15 (PMC2099483; doi:10.1186/1471-2105-8-S7-S15)

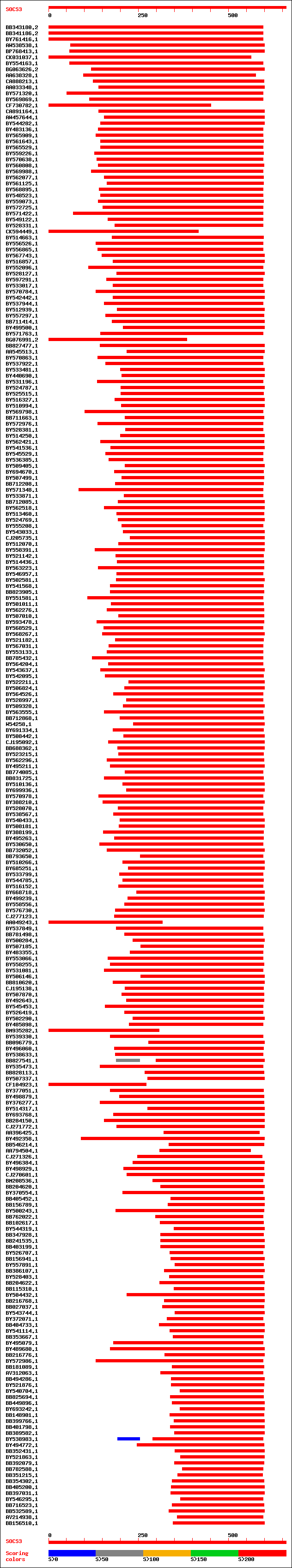

Supplement: Additional file 1 — Graphical overview of the BLAST alignment of mouse genome fragment containing SOCS3 sequence and homologous mouse ESTs. [file 1471-2105-8-S7-S15-S1.doc]
